# Work-related Musculoskeletal Disorders in Korea Provoked by Workers’ Collective Compensation Claims against Work Intensification

**DOI:** 10.1186/2052-4374-26-19

**Published:** 2014-07-29

**Authors:** Dongmug Kang, Youngki Kim, Young-Il Lee, Sangbaek Koh, Inah Kim, Hoonkoo Lee

**Affiliations:** 1 Department of Occupational and Environmental Medicine, Pusan National University, Yangsan Hospital, Yangsan 626-770, South Korea; 2 Department of Occupational and Environmental Medicine, Yonsei University, Wonju Severance Christian Hospital, Wonju, South Korea; 3 Graduate School of Public Health, Yonsei University, Seoul, South Korea; 4 Korean Institute of Labor Safety and Health, Seoul, South Korea

**Keywords:** Musculoskeletal, Neoliberalism, Risk assessment, Compensation, Work intensification, Struggle, Campaign, Globalization

## Abstract

This article presents the process of workers’ problems with work related musculoskeletal disorders (WMSDs), the introduction of risk assessments (RA) for their prevention, and the consequences of this process in Korea. In 1997, economic crisis caused a rapid increase of massive layoffs, worker dispatch system introduction, job insecurity, and use of irregular workers resulting in work intensification. Work intensification increased WMSDs, which created massive workers’ compensation collective claims. Workers argued for the reduction of work intensity. The RAs introduced as a consequence of the workers’ struggle is unique in the world. Whereas these RAs were expected to play a pivotal role in WMSDs prevention, they dis not due to workers’ lack of engagement after the compensation struggle. In fact, changes in the compensation judgment system and criteria have resulted in lower compensation approval rates leading to lower workers’ compensation claims. The Korean experience provides insight into WMSDs causes in a globalized world. In such a the globalized world, work intensification as the result of work flexibility could be an international trend.

## Introduction

Work-related musculoskeletal disorders (WMSDs) are about 70% of the among compensated occupational diseases in Korea in recent years [1], and their economic effects have cost 7 billion dollars [2]. Non-accidental WMSDs are relatively new work-related disorders in Korea, that were rarely compensated before the 1990s. Two compensated cases were reported in 1993, and the cases increased up to 345 cases in 1996. The history of research on WMSDs is similar. The first study was published in 1989 with self-reported symptoms among international telephone operators [3], and other studies were published after the mid 1990s [4-8].

Compensated WMSDs decreased in 1997 to 133 cases, and in 1998 to 72 cases. These decreases might be explained by unstable employment caused by a national economic crisis. After the recovery from the economic crisis in 2000, the numbers surpassed expectation. This explosive expansion of compensated WMSDs was a greater than natural increase. The Korean Confederation of Trade Unions (KCTU) recognized WMSDs cause as a consequence of globalization resulting in work intensification, and organized collective compensation claims. Although this situation shows unique features of socialization of WMSDs problems, there has been no analysis and publication yet.

This paper presents the earlier history of WMSDs, the process of collective compensation claims, and their results.

## Review

### Beginning of compensated WMSDs in Korea in the 1980s

Although many accidental MSDs had been compensated, there were no recorded compensated non-accidental WMSDs in Korea before the 1980s. The first recorded compensated WMSDs were 20 female wrist ganglion cases at Now Precision Industry, located in Guro, Seoul in 1988 [9]. Then, 6 lumbar disc herniation cases were compensated from Namil Metal, located in Incheon in 1989 [10]. After these 6 cases, an epidemiologic investigation was conducted [11]. This epidemiologic investigation was the first one for WMSDs in Korea. As a result of the investigation, 156 compensated back problems in factories from 1985–1988 were revealed. These factory cases began compensated WMSDs in Korea.

### Expansion and collapse of compensated WMSDs in the 1990s

Compensated WMSDs increase from the early 1990s until 1996, and then decreased after 1997 until 1999 (Table 1). Expansion of compensated WMSDs in the early and middle 1990s was partially caused by collective claim applications by labor unions. The labor union of Mando Machine claimed neck-shoulder-arm disorders & back problems as work-related disorders to the Korean Workers’ Compensation and Welfare Service (COMWEL) in 1994. Kia Automobile’s labor union collectively claimed WMSDs. Interestingly, the union argued WMSDs resulted from work process changes due to work intensification. Collective claims from Korea Telecom (KT) with neck-shoulder-arm disorders among telephone operators occurred in 1995 and 1996 with 345 resulting compensated cases in 1996 [12]. As a consequence of KT’s collective claim, “Guidelines for VDT workers” by the Ministry of Labor and Proclamation of MoL 98–15 “Guidelines for repetitive workers” were issued. After 1996, compensated WMSDs had decreased in 1997 to 133 cases, and in 1998 to 72 cases. These decreases might be explained by unstable employment due to a national economic crisis.

**Table 1:** **Compensated work-related musculoskeletal disorders in 1990s**[13]

**Year**	**Total compensated work related disorders, n (%)**	**Compensated work-related musculo-skeletal disorders, n (%)**
1993	1,413 (100.0)	2 (0.1)
1994	918 (100.0)	20 (2.2)
1995	1,120 (100.0)	128 (11.4)
1996	1,529 (100.0)	345 (22.6)
1997	1,424 (100.0)	133 (9.3)
1998	1,288 (100.0)	72 (6.6)

### Neo-liberalization and work intensification in the late 1990s

**Figure 1:**
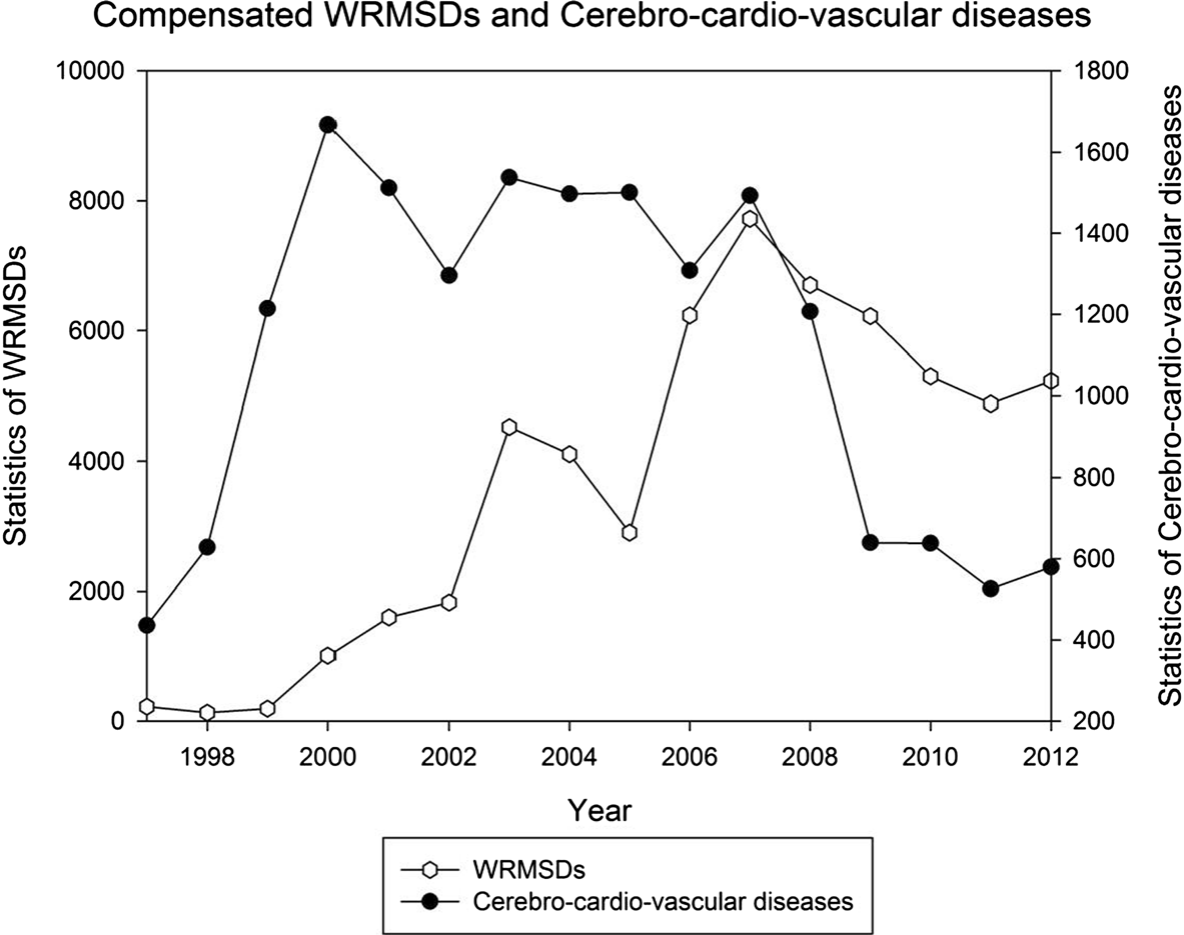
**The proportion of irregular workers in Korea **[14]**.**

Korea experienced an economic crisis from lack of foreign currency in 1997. The Korean government requested urgent fund aid from the International Monetary Fund (IMF). IMF demanded pre-requisite conditions for the aid including legalization of clearance layoffs for the aid. After legalization of clearance layoffs and the labor dispatch system by the National Assembly despite workers’ protests, many companies conducted clearance layoffs, increasing irregular workers. Figure 1 shows irregular workers statistics by MoL and Korea Labour and Society Institute (KLSI) based on the national statistical bureau, KOSTAT. Both lines on the graph present rapid increases of irregular workers after the economic crisis.

These serial situations between the end of 1990s and the beginning of the 2000s can be understood by the introduction of neo-liberalization [15]. Neo-liberalization is defined by free capital movement across the board and globalization of the labor market [16]. This definition could be further specified by deregulation of barrier to profit, disinvestment in welfare states, reduced impact of trade unions, and privatization of the public sectors [17].

**Figure 2:**
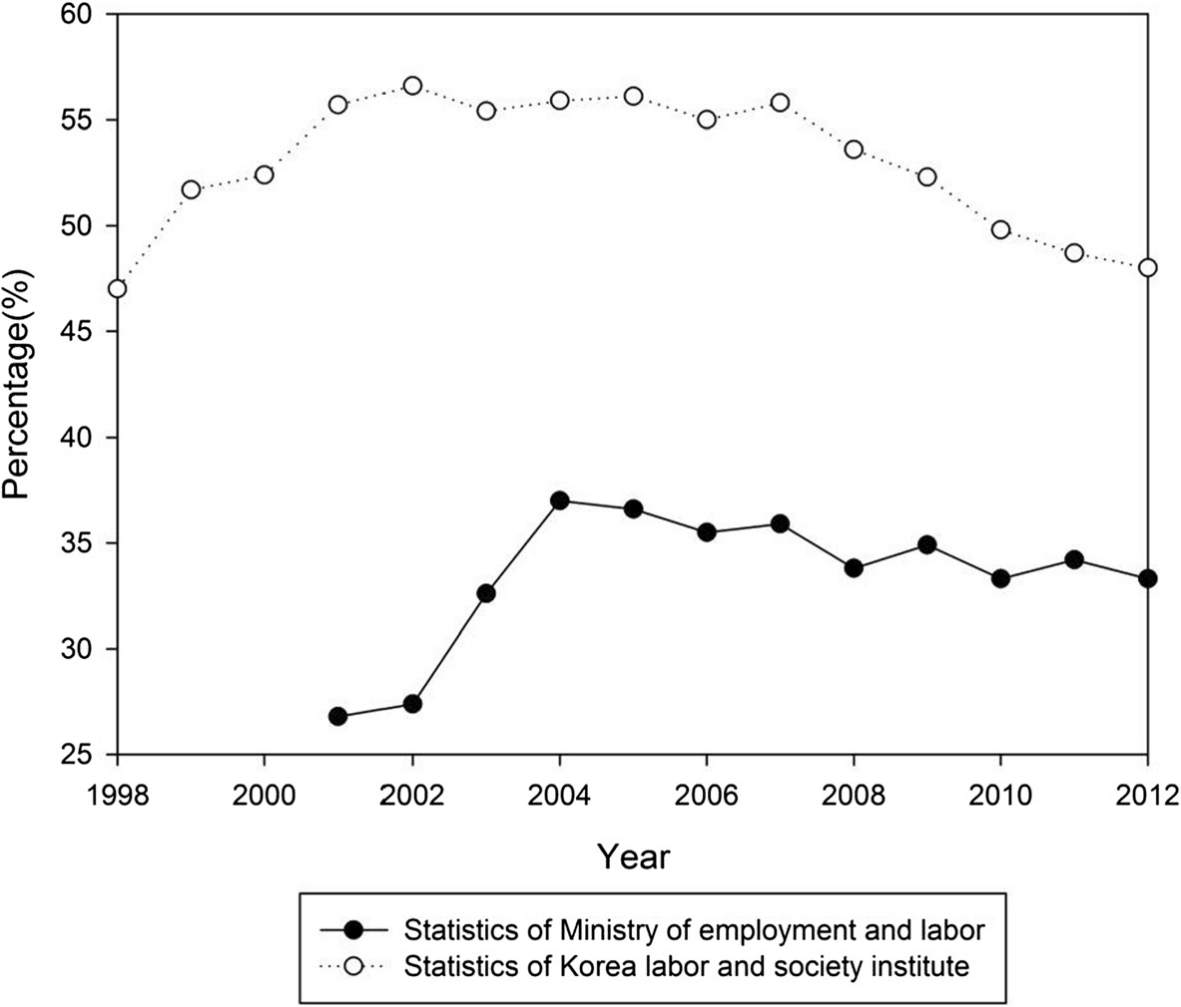
Conceptual framework of work intensification by work process change.

Workers’ complaints had increased because of job insecurity caused by usual layoffs, increases of irregular workers, deterioration of working conditions, and the regression of occupational health and safety systems during the economic crisis. Reduction of workers’ numbers because of layoff resulted in work intensification after the recovery from economic crisis. Some of labor unions recognized WMSDs as the result of work intensification. They realized that working hour elongation and work density intensification from the neoliberalism of globalization were causes of WMSDs. This realization resulted in a questionnaire to evaluate work intensification as a result of research conducted at Daewoo shipbuilding [18]. The conceptual framework of work intensifications as causes of WMSDs is shown in Figure 2. Neo-liberalized globalization generally resulted in flexibility in wages, employment types, working hours, working density, and employer-employee relationships, which led to changes in working structures and working conditions because of changes in the labor market [19]. Increases in work flexibility pursue maximization of profit rates by increasing absolute work intensity (elongation of working hours) and relative work intensity (work density intensification). Workers argued WMSDs were due to these work intensifications. Considering Korea was the most flexible of 18 countries, the impacts of neo-liberalization on the musculoskeletal system would be larger than other OECD countries [20]. Many studies using the work intensification questionnaire showed a similar relationship between work intensification and WMSDs to Korea [21-23].

### Workers’ struggles for WMSDs compensation

**Figure 3:**
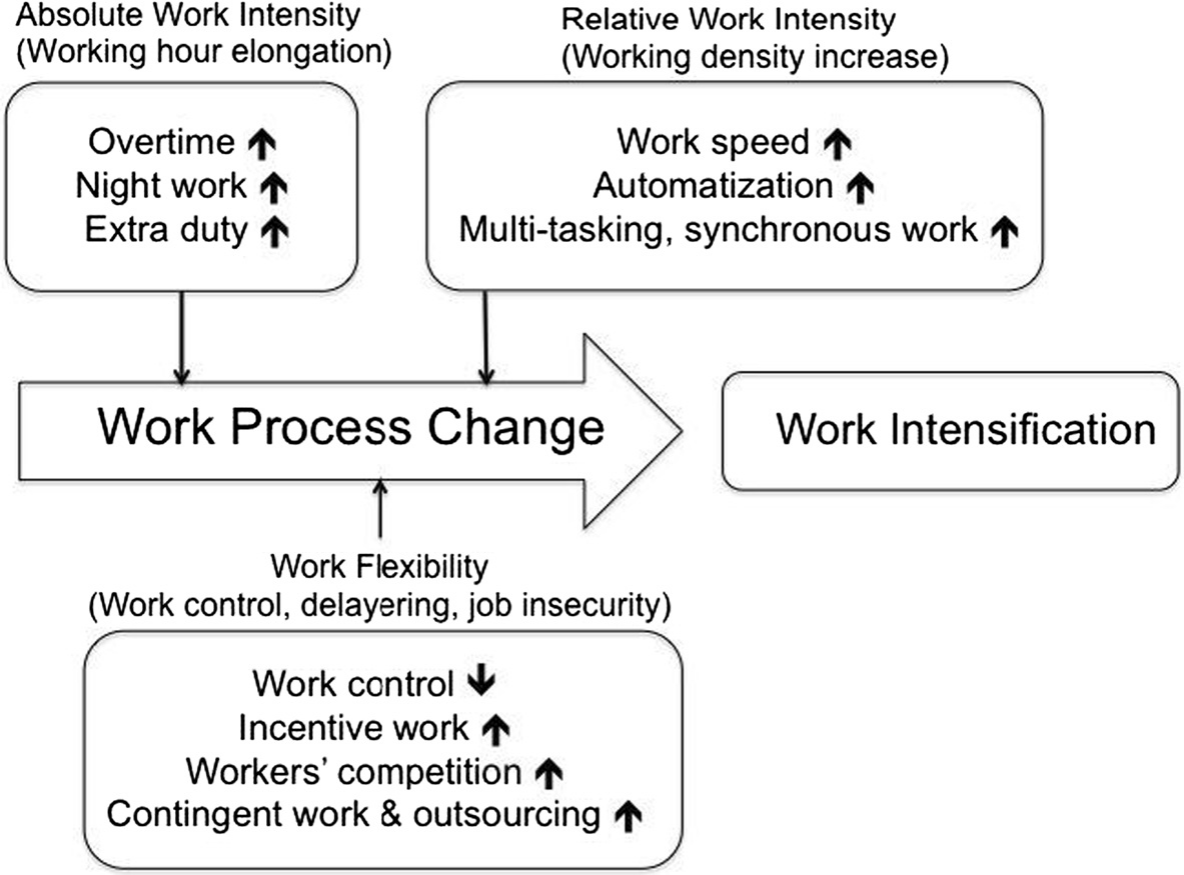
**Statistics of compensated WMSDs and cerebro-cardio-vascular diseases **[24]**.**

**Figure 4:**
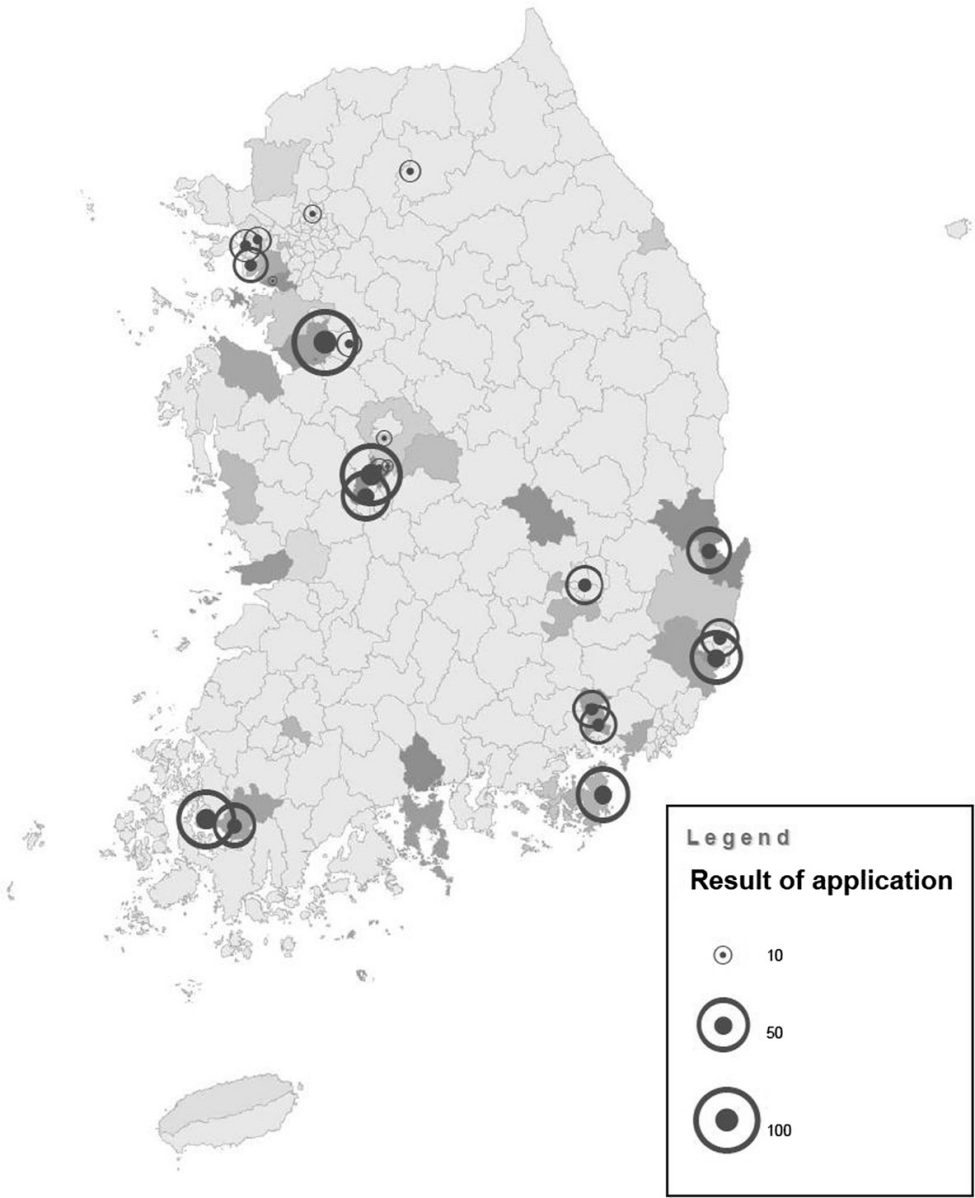
**Distribution of collective compensation claims.** Rounds indicate magnitude of the compensation cases shown in Table 2. Black areas indicate industrial complex.

Figure 3 shows the compensation statistics of WMSDs after the economic crisis. Given no change of compensation schemes and criteria, there were specific causes of explosive increases in compensation numbers. This explosion might reflect the workers’ massive struggle. KCTU started field surveys in 2000 to evaluate companies’ WMSDs situations for those reasons. Hyundai Precision Industry’s labor union was the first to collectively claimed 64 cases and 50 cases were approved compensation. In 2002, Daewoo Shipbuilding’s labor union conducted research on the social impact of the struggle. As a result of the research, the treatment needs of more than 400 workers were recognized. Collective claims of 87 cases resulted in 76 approved compensation. After this, about 41 labor unions struggled for WMSDs’ compensation (Table 2). The compensation claims rose from all around the nation, especially in industrialized sections (Figure 4). However, there were also claims from non-industrialized portions and non-metal workers, such as the hospital and food sectors.

**Table 2:** **Chronicle of massive workers’ compensation claims for musculoskeletal disorders during 2002–2004 in Korea**[25]

**Year**	**Month**	**Company name**	**Approval**
2000	Oct	Hyundai precision	50
2002	Mar	Daewoo shipbuilding	76
	Jul	Casco et al. in Kyung-nam area	32
	Jul	Hallaviston climate control	11
	Aug	VDO, Kamko et al. in Daejeon and Chung-buk area	10
	Sep	Hallaviston climate control	5
	Nov	Daewoo automobile	26
2003	Jan	Samho heavy industry	33
	Jan	Doowon precision	20
	Jan	Korea piston ring	7
	Mar	Daewoo heavy industry and Machinary	27
	Mar	Open SE, Pulmuone	26
	Apr	Hyundai automobile	32
	May	INI steel	29
	May	Samho shipbuilding	89
	Jun	14 companies in Chung-nam area	94
	Jul	9 companies in Pohang area	46
	Jul	Ssangyongautomobil	109
	Aug	5 companies in Chung-nam area	49
2004	Feb	Dimos	6
	Jun	Kyungpook university hospital	32
	Oct	Rotem	32

The major demands of workers were as follows. First, proper treatment and compensation were to be provided thought the entire approval of all claims. Second, work intensity causing WMSDs needed to decrease. Third, WMSDs prevention required legalization. The demands to decrease work intensity included increasing of employees, shortening working hours, reducing of production goals, and improving of working conditions. Successful cases, such as Doowon Precision Co., resulted in increased of employee numbers, decreased of production, improvement of working design, and decreased of work intensity.

### Risk assessment for WMSDs as a result of workers’ struggles

The workers’ struggles caused many things. Major outcomes were the recognition of the work- relatedness of musculoskeletal disorders and the possibility of compensation. This expenses workers’ opportunities to be treated. This is an important point in a situation like Korea’s. Because there is no disease pension in Korea, occupational compensation should increase opportunities for treatment. This opportunity increased compensation claims after the workers’ struggles. A second outcome was the introduction of mandatory risk assessment (RA) for WMSDs. This nationwide mandatory RA is focuses on prevention, which is unique in the world. The major contents of this RA is to investigate risk factors and symptom complaints subject, and to report them to the MoL. Because personnel in each company did not have knowledge and skills for the RAs at the beginning of the regulation (year 2004), contracted experts such as ergonomists and/or occupational physicians from outside organizations had usually conducted the RAs. After the regulation has became official, inside personnel conducted the RAs. Meanwhile labor unions of 9 companies in the Changwon area conducted RAs with their own research team composed of field workers under the motto of “field workers are experts who know the real world”. This grassroots research was conducted in other areas such as Pohang, Chung-Nam, Daejeon, and Chung-Buk with research method of participatory action oriented research methods.

### Work intensification as a fundamental cause of WMSDs

WMSDs problems in Korea have unique features that has been raised by workers. The work intensity issue argued as a cause of WMSD brought new insight. Although European countries have long histories of workers’ struggles for occupational health and safety, harsh and organized struggle on specific issues by workers in Korea is hard to find in the world [26]. These serial situation after the end of 1990s in Korea could be the result of neo-liberalization. The relationship between neo-liberalization, working condition changes, and health has been reported in researches. Kristensen et al. recognized work intensification by work speed increase and working hour elongation in the globalized labor market, and considered to introduced a measurement tool for work intensification [27]. Helenice reported the occupational injury increases due to 3.7% annual work force reduction with annual production per capita increases during 1991 – 1995 [28]. Kuorinka argued for the necessity of new prevention strategies for WMSDs in a “fast production method” society which could be characterized by flexible production systems resulting in subcontracting, reduction of production cycles, and lean production [29].

### After workers struggle

Collective compensation claims by workers during 2002 to 2004 led the MoL to introduce RAs in 2004 despite business opposition. However, the initiative on WMSDs issues begun by workers has turned to government and entrepreneurs. Although some labor unions took advantage of RAs, employers in majority companies have led the process of reducing the effects of work environment on improvement and prevention of WMSDs. The impact of labor unions and workers on the WMSDs issue has decreased during this process. Meanwhile, COMWEL changed the compensation criteria reducing approval rates of compensation, which made it difficult for each worker to apply for compensation claims. Also, large industries have facilitated on-site medical clinics, physiotherapy treatment units, and exercise treatment units to treat WMSDs in factories. They have also provided medical subsidies. All these efforts by companies make workers to avoid compensation claims. In the smaller companies which do not have labor unions, WMSDs complaints might act as a risk factor of job insecurity.

The RAs introduced by workers struggles would have been an important tool for WMSDs prevention, if they had worked properly. This RAs are in danger of being abandoned because of business pressure. Although it is an obligatory legal system, 20.4% and 72.1% of companies with more and less than 50 employees respectively did not take this RAs [30]. Also, companies where RAs were taken seldom improved working conditions, which means this system is just a compliance exercise [31]. This situation might be the consequence of labors focus on compensation and neglect of prevention such as RAs. Also, interest from workers could have as the result of insignificant working condition improvements after RAs. This phenomenon paradoxically shows workers’ roles in successful occupational safety and health systems. WMSDs problems in Korea have been brought to light by the pioneering activity of workers. This very meaningful event in the world suggests macro ergonomic issues as well as micro ergonomic issues as risk factors of WMSDs.

## Conclusions

WMSDs problem in Korea had the unique feature of being brought by workers. Furthermore the workers’ argument of work intensification as a cause of WMSDs resulting from work flexibility because of neo-liberalized globalization provides thoughtful insight. Whereas the mandatory RAs for prevention of WMSDs as a result of workers’ protests are unparalleled in the world history, they are in danger of abandonment because of mean effectiveness resulting from worker follow up. This process presents workers’ roles in occupational safety and health areas. In the globalized world, work intensification as a result of work flexibility could be an international trend.

## Competing interests

The authors disclose have no conflicts of interests.

## Authors’ contributions

DMK planed the study, structured the statements and translated into English. YKK and YIL reviewed and wrote manuscript. SBK, IAK, HKL reviewed and corrected manuscript. All authors read and approved the final manuscript.
